# The effect of iloprost and sildenafil, alone and in combination, on myocardial ischaemia and nitric oxide and irisin levels

**DOI:** 10.5830/CVJA-2017-025

**Published:** 2017

**Authors:** Aydin Suna, Azboy Davut, Temizturk Zeki, Aydin Suna, Kuloglu Tuncay, Aydin Suleyman, Yardim Meltem, Kemal Kalkan Ali, Nesimi Eren Mehmet

**Affiliations:** Department of Cardiovascular Surgery, Elazig Education and Research Hospital, Health Science University, Elazig, Turkey; Department of Cardiovascular Surgery, Elazig Education and Research Hospital, Health Science University, Elazig, Turkey; Department of Cardiovascular Surgery, Elazig Education and Research Hospital, Health Science University, Elazig, Turkey; Department of Anatomy, School of Medicine, Firat University, Elazig, Turkey; Department of Histology and Embryology, School of Medicine, Firat University, Elazig, Turkey; Department of Medical Biochemistry (Firat Hormones Research Group), School of Medicine, Firat University, Elazig, Turkey; Department of Medical Biochemistry (Firat Hormones Research Group), School of Medicine, Firat University, Elazig, Turkey; Department of Cardiology, Education and Research Hospital, Istanbul, Turkey; Department of Cardiovascular Surgery, School of Medicine, Dicle University, Diyarbakir, Turkey

**Keywords:** iloprost, sildenafil, nitric oxide, irisin, myocardial ischaemia–reperfusion

## Abstract

**Aim:**

Insufficient oxygen supply to organs and tissues due to reduced arterial or venous blood flow results in ischaemia, during which, although ATP production stops, AMP and adenosine continue to be produced from ATP. The fate of irisin, which causes the production of heat instead of ATP during ischaemia, is unknown. Iloprost and sildenafil are two pharmaceutical agents that mediate the resumption of reperfusion (blood supply) via vasodilatation during ischaemic conditions. Our study aimed to explore the effects of iloprost and sildenafil on irisin levels in the heart, liver and kidney tissues and whether these pharmaceutical agents had any impact on serum irisin and nitric oxide levels in rats with induced experimental myocardial ischaemia.

**Methods:**

The study included adult male Sprague-Dawley rats aged 10 months and weighing between 250 and 280 g. The animals were randomly allocated to eight groups, with five rats in each group. The groups were: sham (control), iloprost (ILO), sildenafil (SIL), ILO + SIL, myocardial ischaemia (MI), MI + ILO, MI + SIL and MI + ILO + SIL. The treatment protocols were implemented before inducing ischaemia, which was done by occluding the left coronary artery with a plastic ligature for 30 minutes. Following the reperfusion procedure, all rats were sacrificed after 24 hours, and their heart, liver and kidney tissues and blood samples were collected for analyses. An immunohistochemical method was used to measure the change in irisin levels, the ELISA method to quantify blood irisin levels, and Griess’ assay to determine nitric oxide (NO) levels in the serum and tissue. Myocardial ischaemia was confirmed based on the results of Masson’s trichrome staining, as well as levels of troponin and creatine kinase MB.

**Results:**

Irisin levels in biological tissue and serum dropped statistically significantly in the ischaemic group (MI), but were restored with ILO and SIL administration. Individual SIL administration was more potently restorative than individual ILO administration or the combined administration of the two agents. NO level, on the other hand, showed the opposite tendency, reaching the highest level in the MI group, and falling with the use of pharmaceutical agents.

**Conclusions:**

Individual or combined administration of ILO and SIL reduced myocardial ischaemia and NO levels, and increased irisin levels. Elevated levels of irisin obtained by drug administration could possibly contribute to accelerated wound recovery by local heat production. Sildenafil was more effective than iloprost in eliminating ischaemia and may be the first choice in offsetting the effects of ischaemia in the future.

## Aim

Myocardial ischaemia impairs the function and survival of cardiac myocytes. Current treatment for this condition is elimination of ischaemia.^1^ Although the use of coronary dilator anti-aggregatory medications to various degrees is the treatment of choice in the elimination of ischaemia,^2^,^3^ surgical coronary bypass methods are also used, as laid down in treatment guidelines.^4^-^6^ Additionally, iloprost is the first line of treatment in occlusions seen in peripheral artery disease.^7^

Iloprost (ILO) is an eicosanoid pharmaceutical agent from the prostacyclin group.8 Currently, it is clinically used to unblock occluded vessels.^9^,^10^ ILO exercises its vasodilatory effect by preventing platelet aggregation.^8^,^11^ Another vasodilatory agent that acts via nitric oxide (NO) is sildenafil (SIL).^12^ NO levels increase during ischaemia.13 During reperfusion, NO levels are elevated,13 caused by the vasodilator effect of SIL.^14^ The increased NO levels combine with a superoxide radical (O2 -) to form a toxic oxygen metabolite, peroxynitrite (ONOO-), which causes damage in the tissues.^15^,^16^ Therefore, elucidation of the changes in NO levels caused by the administration of ILO and SIL, which were used for the reperfusion of tissues, could help explain the mechanisms underlying this process.

At present, sildenafil is employed to correct erectile dysfunction.12Since it acts as a vasodilator, it can serve as a therapeutic agentduring ischaemia.^14^ Vasodilatation enhances oxygenation andtherefore mediates in the elimination of ischaemia and increasesadenosine triphosphate (ATP) formation. It is established thataerobic ATP formation is blocked in hypoxic states. Therefore,ischaemia leads to a decrease in ATP production.^17^ Anothermolecule that causes a reduction in ATP levels is irisin.^18^,^19^ Byincreasing the amount of uncoupling proteins, this molecule leadsto the release of heat rather than ATP from molecules.^18^

Since ILO,^7^ used in ischaemic peripheral artery disease, and SIL,^12^ used in erectile dysfunction, increase oxygenation through vasodilatation, the tissues recovered from ischaemia would theoretically be expected to have elevated ATP levels. On the other hand, in the presence of irisin, heat production would increase through uncoupling of proteins and cause a decrease in ATP production.^18^,^19^ Therefore there seems to be an obvious correlation between the treatment of ischaemic tissue with ILO and SIL, and irisin levels.

Furthermore, myocardial ischaemia does not only affect heart tissue. It was reported in previous studies that myocardial ischaemia could directly impact on kidney tissue,^20^ which is an excretory organ, and the liver,21 where glycogenesis takes place. In addition, there is an increased need for energy (glucose) during ischaemic conditions. It was reported that irisin inhibited glycogenesis, or impeded the production of glucose.^22^

Therefore the aim of this study was to determine the change in irisin level in tissues with increased energy needs under ischaemic conditions. Our principal objectives were to explore (1) whether ILO and SIL played a part in recovery after myocardial injury and how they changed irisin expression in experimentally induced myocardial ischaemia–reperfusion; (2) whether ILO, SIL, or a combination of both were more efficient in the treatment of ischaemic injury; (3) how NO levels were altered in response to these therapeutic agents; (4) whether irisin, which causes metabolisation of ATP, was down- or upregulated in tissues with an increased need for ATP, as in the case of ischaemia; and (5) how ILO and SIL treatment affected irisin expression in heart, liver and kidney tissues under ischaemic conditions.

## Methods

All protocols of the animal experiments were approved (date 5.2.2014, decision no: 35) by the Animal Ethics Committee (FUAEC) in accordance with the policy of the European convention for the protection of vertebrate animals. The study included adult male Sprague-Dawley rats aged 10 months and weighing between 250 and 280 g. The rats were randomly divided into the following groups: control group (sham: no procedure to be applied, only physiological serum administered), ILO, SIL, ILO + SIL, myocardial ischaemia (MI), MI + ILO, MI + SIL and MI + ILO + SIL. Each group contained five rats.

Ischaemia was induced by left coronary artery ligation, as described previously.^23^ In rat experiments, sildenafil citrate (Viagra) is usually used in the 1–2.5-mg/kg dose range,^24^,^25^ and ILO in the 0.2–2-μg/kg range.^26^,^27^ In this study, 2 mg/kg sildenafil citrate was administered to the SIL group and 1 μg/kg to the ILO group via the intraperitoneal route before the induction of ischaemia–reperfusion, as described previously by Harada et al.^28^ A 30-minute occlusion was then induced using a plastic ligature, as described previously.^29^ After the ligature was released, blood flow was visually confirmed. All rats were sacrificed at 24 hours following the reperfusion procedure.

Blood samples were collected as described for previous experiments,30 centrifuged at 4 000 rpm and stored at –80°C until the irisin analysis. Glucose, creatine kinase (CK), creatine kinase MB (CKMB) and troponin I on the other hand, were analysed without delay on an auto-analyser. Heart, liver and kidney tissue was fixed in 10% formaldehyde solution and stored for immunohistochemical analysis. The remaining heart, liver and kidney tissue, after the wet weight was determined, were homogenised and the supernatants were stored at –80°C for NO analysis.

As its half life is short, it is difficult to directly analyse NO. For NO measurements, its stable end-products, nitrite and nitrate, are quantified in tissues with a spectrophotometric method. This method is based on the principle of measuring the absorbance at 545 nm of the complex formed when nitrate is reduced to nitrite in the presence of nitrate reductase enzyme, and the resulting nitrite reacts with sulfanylamide and N-ethylendiamin.^31^

Serum irisin levels were determined using the ELISA method, following the catalogue guidelines provided by the manufacturing firm.^32^ The kit was reported to have a minimum irisin detection limit of 1.29 ng/ml and minimal cross-reactivity (~9%) with fibronectin type III domain-containing protein 5 (FNDC5). In our laboratory results, we found an intra-assay value of 8% and inter-assay value of 10%.

Histopathological examinations were carried out using the triphenyl tetrazolium chloride method to identify the damage to the myocardium and other tissues, as described previously.^23^ Myocardial injury was assessed according to the semi-quantitative method of Miller et al.^33^ The Abc immunohistochemical method of Hsu et al.34 was used to determine the distribution of irisin expression in the myocardium and other tissues.^34^

## Statistical analysis

The extent of the damage in the myocardium and other tissues was determined using the Student’s t-test. SPSS 22 software was employed in all statistical analyses. Level of statistical significance was determined at a p-value of 0.05.

## Results

**Fig. 1:**
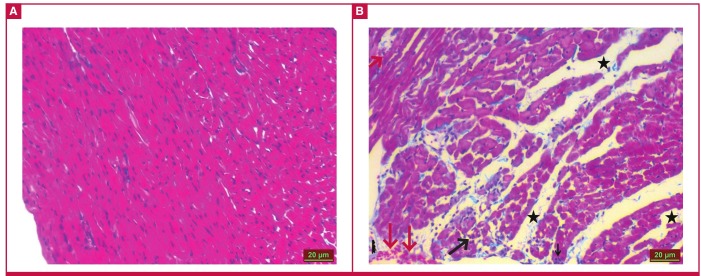
Ischaemic and control heart tissues after Masson’s trichrome staining. The ischaemic group shows an increase in inflammatory cells (black arrow), congestion (red arrow), impairment of tissue integrity and oedema (black asterisk).

Masson’s trichrome staining results under light microscopy showed that the heart tissue of the control group had a normal appearance (Fig. 1A). The MI group, however, showed an increase in inflammatory cells (black arrow), congestion (red arrow), impairment of tissue integrity and oedema (Fig. 1B). Data from the statistical analysis of histopathological changes in the MI group are given in Table 1.

**Table 1:** Histological scores in the heart tissue of rats with induced cardiac ischaemia

	*Inflammatory Cells*	*Congestion*	*Fibrosis*	*Oedema*	*Tissue Integrity*	*Necrosis*
Control	0 ± 00	0 ± 00	0 ± 00	0 ± 00	0 ± 00	0 ± 00
MI	2.33 ± 0.51^a^	2.50 ± 0.83^a^	0 ± 00	3.16 ± 0.40^a^	2.83 ± 0.98^a^	0 ± 00

MI: myocardial ischaemia–reperfusion; aIn comparison with the control group, p < 0.05.

**Fig. 2:**
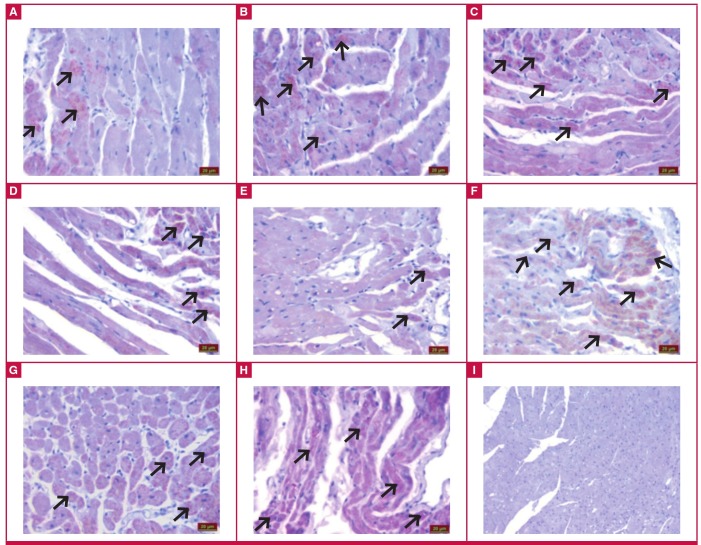
Irisin immunoreactivity in the heart tissues with iloprost (ILO) and sildenafil (SIL) administration in cardiac ischaemia (MI) induced by left coronary artery ligation. Control (A), ILO (B), SIL (C), ILO + SIL (D), MI + ILO (F), MI + SIL (G), MI + ILO + SIL (H), and negative control; no irisin immunoreactivity (I).

Evaluation under the light microscope of immunohistochemical staining revealed irisin immunoreactivity in the muscle cells of the cardiac tissue (black arrow). The control (Fig. 2A), ILO (Fig. 2B), SIL (Fig. 2C) and ILO + SIL (Fig. 2D) groups had similar irisin immunoreactivity. Compared to the control group, the MI group (Fig. 2E) had statistically significantly reduced irisin immunoreactivity (p < 0.05). Relative to the MI group, MI + ILO (Fig. 2F), MI + SIL (Fig. 2G) and MI + ILO + SIL (Fig. 2H) all showed elevated irisin immunoreactivity, similar to that of the control group.

**Fig. 3:**
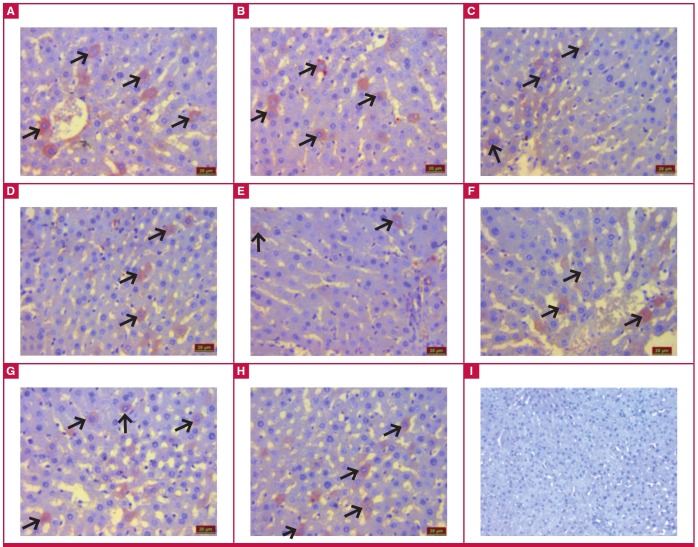
Irisin immunoreactivity after iloprost (ILO) and sildenafil (SIL) administration to hepatic tissues with cardiac ischaemia. Control (A), ILO (B), SIL (C), ILO + SIL (D), MI + ILO (F), MI + SIL (G), MI + ILO + SIL (H) and negative control; no irisin immunoreactivity (I).

Irisin immunoreactivity (black arrow) was also observed in the hepatocytes of the liver tissue in all groups. Irisin immunoreactivity was similar in the control (Fig. 3A), ILO (Fig. 3B), SIL (Fig. 3C) and ILO + SIL (Fig. 3D) groups. However, relative to the control group, the MI group had statistically significantly reduced irisin immunoreactivity (p < 0.05), while the MI + ILO (Fig. 3F), MI + SIL (Fig. 3G) and MI + ILO + SIL (Fig. 3H) groups had significantly increased irisin immunoreactivity, similar to that in the control group.

**Fig. 4:**
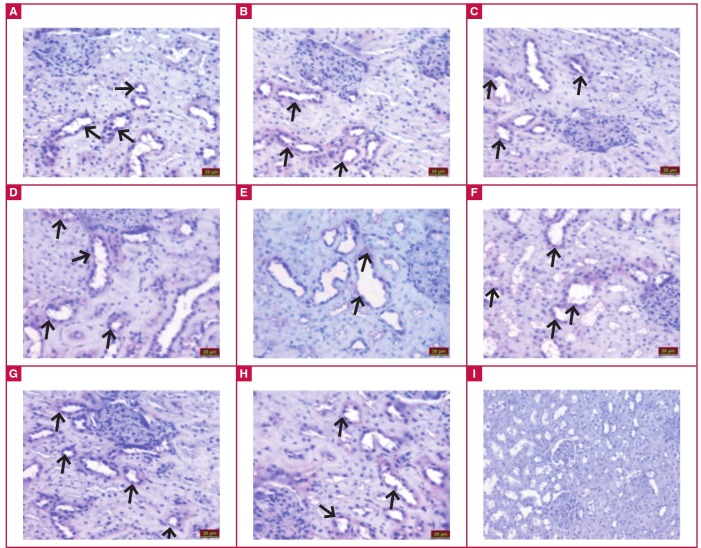
Irisin immunoreactivity after iloprost (ILO) and sildenafil (SIL) administration to renal tissues with cardiac ischaemia. Control (A), ILO (B), SIL (C), ILO + SIL (D), MI + ILO (F), MI + SIL (G), MI + ILO + SIL (H) and negative control; no irisin immunoreactivity (I).

Examination under the light microscope of immunohistochemical staining to detect irisin immunoreactivity showed that the tubule cells in the renal cortex of the kidney of all groups had irisin immunoreactivity (black arrow). Irisin immunoreactivity was similar in the control (Fig 4A), ILO (Fig. 4B), SIL (Fig. 4C) and ILO + SIL (Fig. 4D) groups. In comparison with the control group, the MI (Fig. 4E) group had a statistically significant decrease in irisin immunoreactivity (p < 0.05). However, the MI + ILO (Fig. 4F), MI + SIL (Fig. 4G), MI + ILO + SIL (Fig. 4H) had a significantly higher irisin immunoreactivity. Table 2 summarises the histological scores of irisin immunoreactivity in all tissues and groups.

**Table 2:** The histological scores pertinent to irisin immunoreactivity in all tissues and groups

	*Heart*	*Liver*	*Kidney*
Control	0.83 ± 0.12	0.69 ± 0.15	0.72 ± 0.15
ILO	0.78 ± 0.09	0.82 ± 0.24	0.75 ± 0.17
SIL	0.74 ± 0.13	0.78 ± 0.18	0.76 ± 0.15
ILO + SIL	0.75 ± 0.12	0.61 ± 0.12	0.65 ± 0.15
MI	0.24 ± 0.05^a^	0.28 ± 0.07^a^	0.26 ± 0.05^a^
MI + ILO	0.76 ± 0.15^b^	0.77 ± 0.12^b^	0.61 ± 0.17^b^
MI + SIL	0.85 ± 0.05^b^	0.68 ± 0.10^b^	0.82 ± 0.13^b^
MI + ILO + SIL	0.73 ± 0.15^b^	0.70 ± 0.14^b^	0.64 ± 0.08^b^

^a^In comparison with the control group, bin comparison with the MI group, p < 0.05.

When serum irisin levels in the ischaemic group were compared to those of the control group, they were found to be significantly lower than the controls. However, the groups to which ILO and SIL were administered, either individually or in combination, had elevated serum irisin levels. Serum irisin levels of the control group and the groups administered ILO and SIL before MI induction were statistically similar.

Furthermore, CK, CKMB, troponin I and NO values measured in the study increased with ischaemia. In addition to the histological findings presented above, elevated CK, CKMB and troponin I levels provided further confirmation of the induction of ischaemia with left coronary artery ligation in this animal experiment (Table 3).

**Table 3:** Changes in the levels of CK, CKMB, troponin I, NO and irisin in serum of rats administered iloprost (ILO) and sildenafil (SIL) for cardiac ischaemia

*Groups*	*CK (IU/l)*	*CKMB (IU/l)*	*Troponin 1 (ng/ml)*	*NO (μmol)*	*Irisin (ng/ml)*
Control	6 ± 1	0.00 ± 0	0.01 ± 0	34 ± 7	16.1 ± 1.7
ILO	6.5 ± 1	0.02 ± 0	0.01 ± 0	41 ± 9	14.9 ± 2.9
SIL	6.9 ± 1	0.00 ± 0	0.00 ± 0	29 ± 6	15.8 ± 2.7
ILO + SIL	7.1 ± 2	0.00 ± 0	0.00 ± 0	31 ± 6	16.2 ± 08
MI	648 ± 146^a^	118 ± 54	4.8 ± 9^a^	135 ± 26	9 ± 2.6^a^
MI + ILO	594 ± 116^b^	82 ± 18^b^	3.9 ± 8^b^	96 ± 24	7.6 ± 2.6^b^
MI + SIL	416 ± 119^b^	64 ± 17^b^	3.1 ± 8^b^	62 ± 11	6.3 ± 2.4
MI + ILO + SIL	577 ± 133^b^	77 ± 17^b^	3.4 ± 9^b^	69 ± 12	6.9 ± 2.6^b^

^a^In comparison with the control group, bin comparison with the MI group, p < 0.05

A comparison of serum and heart tissue NO levels between the ischaemic and control groups demonstrated that the former had statistically significantly higher NO levels (Table 3). However, groups to which ILO and SIL were administered, either individually or in combination after the induction of ischaemia, had lower serum and heart tissue irisin levels. NO levels in the serum and heart tissue of the control group and groups administered ILO and SIL before MI induction were statistically similar.

Table 4 summarises NO changes in the heart, liver and kidney tissue supernatants of rats administered ILO and SIL in myocardial ischaemia–reperfusion. There was a negative correlation between serum NO (r = –0.73; p < 0.005) and serum irisin levels (r = –0.52; p < 0.005) of the groups, and positive correlations between the serum and tissue NO levels (r = 0.64; p < 0.005) and myocardial infarct markers [CK (r = 0.49; p < 0.005), CKMB (r = 0.56; p < 0.005) and troponin I (r = 0.66; p < 0.005)]. These correlations either disappeared or were reversed with the administration of ILO and SIL.

**Table 4:** Nitric oxide (μmol/g wet weight) changes in the heart, hepatic and kidney tissue supernatants of rats administered iloprost (ILO) and sildenafil (SIL) in myocardial ischaemia–reperfusion (MI)

	*Heart*	*Liver*	*Kidney*
Control	76 ± 17	54 ± 11	24 ± 7
ILO	85 ± 16	61 ± 13	32 ± 8
SIL	69 ± 14	48 ± 9	19 ± 6
ILO + SIL	71 ± 12	56 ± 10	22 ± 5
MI	248 ± 46^a^	125 ± 29^a^	106 ± 19^a^
MI + ILO	186 ± 32^b^	82 ± 18^b^	73 ± 18^b^
MI + SIL	106 ± 18^b^	64 ± 17^b^	41 ± 9^b^
MI + ILO + SIL	119 ± 21^b^	77 ± 17^b^	59 ± 10^b^

^a^In comparison with the control group, bin comparison with the MI group, p < 0.05.

## Discussion

Disruption of the arterial or venous blood flow in biological systems (hypoxia) causes inadequate perfusion of the organ or tissues, resulting in generalised cell injury or cell death.^5^ Ischaemia–reperfusion injury in the heart tissue causes myocardial stunning, reperfusion arrhythmias, necrosis in the myocytes, as well as coronary endothelial and microvascular dysfunction.^29^ Ischaemia–reperfusion injury in the heart also affects the renal tissues,^20^ and the hepatic tissues where gluconeogenesis takes place.^21^ In this study, ischaemia was induced by left coronary artery ligation, and the roles of ILO and SIL in tissue reperfusion, and their effect on the fate of irisin, which functions in heat regulation, were examined.

The induction of ischaemia by left coronary artery ligation was confirmed by Masson’s trichrome staining of the heart tissue. Light microscopy of the cells showed that the control heart tissues had a normal appearance, while the MI group was characterised by an increase in inflammatory cells, congestion, impairment of tissue integrity and oedema. This resulted from the interruption of ATP production. However, since ATP catabolism continues, AMP and adenosine were formed from ATP. The depletion of cellular energy reserves and the accumulation of toxic metabolites due to ischaemia led to an increase in inflammatory cells, congestion, and finally cell death.^35^ The presence of ischaemia was also confirmed by the levels of CK, CKMB and troponin I, which increased dramatically during myocardial ischaemia, compared to the control levels. The levels of CK, CKMB and troponin I in our study were similar to those found in previous animal studies, where myocardial ischaemia was induced with isoproterenol.^35^

When irisin levels in the ischaemic groups were compared to those in the control group, irisin was statistically significantly lower in the serum, and individual or combined administration of ILO and SIL restored irisin serum concentrations. The ischaemia-associated decrease in irisin concentrations in biological systems may be attributed to increased demand for ATP in the tissues, as irisin elevates the levels of uncoupling proteins, which in turn causes increased heat production in the cells, rather than increased ATP production.^18^,^19^,^36^

However, there is a need for much greater amounts of ATP to reduce the amount of cell injury and death resulting from hypoxia.^17^ Therefore the heart, liver and kidney tissues may have limited their irisin production for the purposes of encouraging cells to produce ATP instead of heat. The decrease in ATP levels during this period indicates that the increase in inflammatory cells, congestion, impairment of tissue integrity and oedema provoked the development of rigor-type contracture.^35^ In the case of ischaemia-associated coronary endothelial dysfunction, the vasodilator response is reduced because the increase in endothelin-1 level, a potent vasoconstrictor, which is formed in the process, causes vasoconstriction and leads to a decrease in blood flow.^37^

In this context, administration of ILO and SIL to the rats individually or in combination enhanced the blood flow by vasodilatation, ensuring re-oxygenation of the cells, and hence increased irisin synthesis. Energy production of the cells during re-oxygenation is probably kept under strict control by irisin (considered as the decisive molecule at the stage of heat or ATP production) via regulation of the flow of oxygen in the electron transport chain in the mitochondrial organ, depending on the need for ATP. Otherwise, the entry of high doses of molecular oxygen into the cell would increase free oxygen radical (FOR) derivatives and cause reperfusion damage.38 This is because about 1–4% of the oxygen intake is used for superoxide anion production and about 20% of the produced superoxide anions is channelled to the cells. This is believed to be directly related to the production of energy molecules or ATP by the cells.

Limited irisin production in biological tissues causes a reduction in serum irisin levels. Given that the source of serum irisin is biological tissue, the decrease in irisin synthesis by the tissues is reflected in serum irisin levels. These data not only support the finding of reduced irisin levels in MI induced by ISO,^35^ but also are consistent with the MI results provided by Aydin et al. in saliva, and by Emanuele et al.^39^,^40^

In our study, ischaemia induced by left coronary artery ligation caused ischaemia–reperfusion injury, not only in the heart, but also in the liver and kidneys. Ischaemic damage inflicted on the liver and kidneys, as in damage to the heart tissue, was reduced by the administration of ILO or SIL, or their combination, relative to the group not administered any drug, and the administration of these drugs also elevated the irisin levels in these tissues. ILO is thought to restore ischaemic injury in the liver and kidneys via its anti-platelet, cytoprotective and fibrinolytic action, and vasodilator effect, while SIL, a specific phosphodiesterase type 5 (PDE 5) inhibitor, is believed to reduce ischaemic injury via its vasodilator effect mediated by NO.^41^

Administration of SIL alone was found to be more effective in reducing ischaemic injury than ILO alone or the combination of both agents. The possible mechanism underlying the potency of SIL is that since it uses NO, the NO produced during ischaemia is reduced in the presence of SIL. Had NO not been depleted or reduced, it would have combined with the superoxide (O2 -) radical produced during reperfusion to form peroxynitrite (ONOO-), a toxic oxygen metabolite, and the resulting ONOO- could have caused damage to the tissues.^15^,^16^ Therefore, a possible reason why SIL was slightly superior to ILO in treating ischaemic injury was that it reduced or depleted the NO formed during ischaemia, as SIL enhances NO-mediated vasodilatation.

In our study, individual administration of SIL (resulting in the lowest NO levels) and combined administration of ILO and SIL were observed to cause a decrease in NO levels in comparison to that found in the ILO alone and ischaemic groups (which had the highest NO levels). Therefore, we believe that this biochemical feature of SIL could be used to eliminate ischaemia–reperfusion injury in the future.

Irisin may contribute to the acceleration of wound recovery, since heat speeds up chemical reactions and enables rapid production of the proteins involved in wound healing, thus promoting rapid recovery. It has recently been reported that wound healing of human umbilical vein endothelial cells (HUVEC) was gradually accelerated in groups treated with 10 and 20 nM irisin at both 12 and 24 hours, via increasing migration and tube formation.^42^ The administration of both drugs in combination did not prove more effective than the individual administration of each. However, when individual administrations of ILO and SIL were compared to one another, SIL was found to be more potent in circumventing ischaemia. One explanation why SIL was more efficient in ischaemia treatment may be that since it is an NO-dependent agent, it can deplete NO arising from ischaemia and therefore reduce the amount of peroxinitrite originating from NO and causing tissue injury.^43^

## Conclusion

In this experimentally induced animal myocardial ischaemia model, administration of ILO and SIL reduced both ischaemia and the release of NO, while elevating irisin levels. Our study showed that even though SIL and ILO have not been routinely used in the management of myocardial ischaemia–reperfusion, both drugs are critical pharmaceutical agents in eliminating tissue ischaemia. Further clinical studies are necessary on patients to elucidate this phenomenon.
